# Feasibility Study of Cord Tissue Derived Mesenchymal Stromal Cells in COVID-19-Related Acute Respiratory Distress Syndrome

**DOI:** 10.1093/stcltm/szad009

**Published:** 2023-03-17

**Authors:** Beth H Shaz, Bryan D Kraft, Jesse D Troy, Emily Poehlein, Lingye Chen, Lynn Cheatham, Raha Manyara, Khalid Hanafy, Linda Brown, Margaret Scott, Ralph Palumbo, Frank Vrionis, Joanne Kurtzberg

**Affiliations:** Marcus Center for Cellular Cures, Duke University, Durham, NC, USA; Department of Medicine, Duke University, Durham, NC, USA; Marcus Center for Cellular Cures, Duke University, Durham, NC, USA; Marcus Center for Cellular Cures, Duke University, Durham, NC, USA; Department of Medicine, Duke University, Durham, NC, USA; Marcus Center for Cellular Cures, Duke University, Durham, NC, USA; Marcus Center for Cellular Cures, Duke University, Durham, NC, USA; Boca Raton Regional Hospital, Boca Raton, FL, USA; Marcus Center for Cellular Cures, Duke University, Durham, NC, USA; Boca Raton Regional Hospital, Boca Raton, FL, USA; Boca Raton Regional Hospital, Boca Raton, FL, USA; Boca Raton Regional Hospital, Boca Raton, FL, USA; Marcus Center for Cellular Cures, Duke University, Durham, NC, USA

**Keywords:** acute respiratory distress syndrome, COVID, mesenchymal stromal cells, cord blood

## Abstract

**Background:**

Treatment options for patients with COVID-19-related acute respiratory distress syndrome (ARDS) are desperately needed. Allogeneic human umbilical cord derived mesenchymal stromal cells (hCT-MSCs) have potential therapeutic benefits in these critically ill patients, but feasibility and safety data are lacking.

**Materials and Methods:**

In this phase I multisite study, 10 patients with COVID-19-related ARDS were treated with 3 daily intravenous infusions of hCT-MSCs (1 million cells/kg, maximum dose 100 million cells). The primary endpoint assessed safety.

**Results:**

Ten patients (7 females, 3 males; median age 62 years (range 39-79)) were enrolled at 2 sites and received a total of 30 doses of study product. The average cell dose was 0.93 cells/kg (range 0.56-1.45 cells/kg and total dose range 55-117 million cells) with 5/30 (17%) of doses lower than intended dose. Average cell viability was 85% (range 63%-99%) with all but one meeting the >70% release criteria. There were no infusion-related reactions or study-related adverse events, 28 non-serious adverse events in 3 unique patients, and 2 serious adverse events in 2 unique patients, which were expected and unrelated to the study product. Five patients died: 3 by day 28 and 5 by day 90 of the study (median 27 days, range 7-76 days). All deaths were determined to be unrelated to the hCT-MSCs.

**Conclusion:**

We were able to collect relevant safety outcomes for the use of hCT-MSCs in patients with COVID-19-related ARDS. Future studies to explore their safety and efficacy are warranted.

## Graphical Abstract

**Graphical Abstract:**
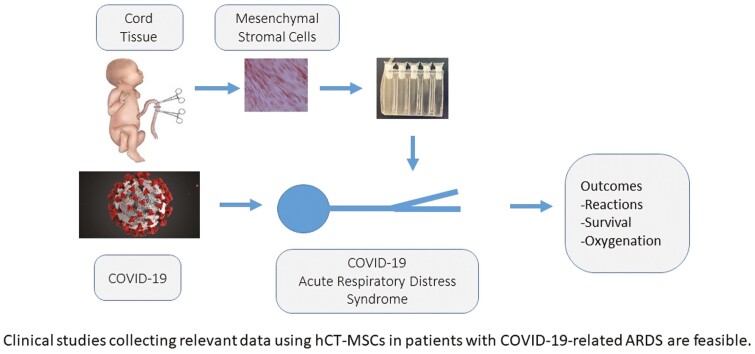

**Significance Statement**

Clinical studies collecting relevant data using hCT-MSCs in patients with COVID-19-related ARDS are feasible.

## Introduction

The coronavirus disease 2019 (COVID-19) is secondary to infection with severe acute respiratory syndrome coronavirus 2 (SARS-CoV-2) resulting in a global pandemic.^1^ In severe cases, COVID-19 leads to acute respiratory distress syndrome (ARDS), thought to be due to direct injury to the lung and hyperinflammatory response.^2^ Treatment recommendations for COVID-19-related-ARDS (COVID-ARDS) have evolved to include remdesivir, dexamethasone, and immunomodulatory therapies; however, there remains no effective treatment for ARDS, resulting in many patients dying from sepsis or multi organ failure.^3,4^ The use of mesenchymal stromal cells (MSC) offers a unique therapeutic option for patients with COVID-ARDS that might shorten time to lung injury resolution.^5^

MSCs from various sources (adipose tissue, bone marrow, and cord tissue) are being studied in clinical trials for patients with COVID-ARDS.^2^ The rationale for this approach is that MSCs have anti-inflammatory properties without apparent toxicity and may provide similar to superior results with less toxicity compared to conventional pharmaceutical treatments. Furthermore, MSCs have been shown to have antimicrobial properties, which, in preclinical models, have been shown to eliminate certain bacteria and mycobacteria from animal and tissue culture models of cystic fibrosis.^6^ MSCs also can differentiate into alveolar epithelial cells in vitro, although their capacity to do this in vivo is not known.^7^ This has led to the hypothesis that MSCs may work through anti-inflammatory, immune-modulatory, and regenerative mechanisms in vivo.^2^

We conducted a feasibility clinical trial, testing the safety of intravenously administered, human cord-tissue derived MSCs (hCT-MSC) for treatment of severe COVID-19 infection characterized by COVID-ARDS. Participants received 3 daily intravenous infusions of hCT-MSCs (1 million cells/kg, maximum dose 100 million cells). This dosing regimen was chosen to support the hypothesis that hCT-MSCs could potentially treat COVID-ARDS if they quickly countered the cytokine release syndrome resulting in ARDS. We now report the results of this study.

## Materials and Methods

### Study Design and Overview

Cord tissue-derived mesenchymal stromal cells in COVID-19- related acute respiratory distress syndrome (MASC) was a phase I, multisite, open-label prospective clinical trial studying the feasibility of 3 hCT-MSC infusions in 10 adults with COVID-ARDS. This manuscript reports the results of the phase I part of a larger 30 patient phase I/IIa multi-center safety study, where the phase II part was a randomized, controlled trial of an additional 20 patients. The phase II portion of the study was modified after the first 10 patients were enrolled to include additional sites, increase the number of patients, and the patients were randomized to hCT-MSCs manufactured by the University of Miami (10 patients) or Duke (10 patients) versus placebo (20 patients). Because of decreased number of patients with COVID-ARDS the phase II study was terminated early due to a lack of enrollment and feasibility. The phase I study was conducted at Duke University and Boca Raton Regional Hospital, Baptist Health South Florida. The hCT-MSCs were manufactured at the Robertson GMP Cell Manufacturing Laboratory at the Marcus Center for Cellular Cures, Duke University, Durham, NC, USA. The study was approved by each clinical site’s institutional review board and registered under investigational new drug (IND) # 19968 and www.clinicaltrials.gov identifier NCT04399889.

Participants were treated with 3 daily intravenous (IV) infusions of allogeneic hCT-MSCs. There was a 3-day hold between the first 3 patients to observe for any infusion-related toxicities. If no toxicity was observed, the next 7 patients could receive treatment without any holds. The Data Safety and Monitoring Board (DSMB) reviewed the safety data after the 10 patients met the 28-day endpoint. Subjects were assessed for eligibility and adverse events by an intensivist. Adverse events (AEs) were documented and reviewed by the study team.

### Participants

Eligible patients were confirmed to have COVID-19 by real-time polymerase chain reaction (RT-PCR). The inclusion criteria were age 18 years or older, adequate contraception use during the treatment and for 6 months post-treatment, and meeting the ARDS Berlin criteria (bilateral opacities on chest imaging consistent with pulmonary edema, need for positive pressure ventilation via endotracheal or tracheostomy tube, PaO_2_/FiO_2_ ratio ≤ 300 mmHg with a minimum of 5 cmH_2_O PEEP, and infiltrates not fully explained by cardiac failure or fluid overload in the physician’s best clinical judgment).^8^ Exclusion criteria were evidence of multiorgan failure involving one or more organs, excluding the lungs (defined by the presence of shock [MAP < 65 mmHg with signs of peripheral hypoperfusion, or continuous infusion of 2 or more vasopressor or inotrope agents to maintain MAP ≥ 65 mmHg], serum bilirubin >10 mg/dL, or platelet count <50 000/μL), acquired or congenital immunodeficiency, history of metastatic cancer diagnosis or treatment within the past year, history of previous treatments with MSCs or other cell therapies, co-enrolled in another IND-sponsored clinical trial for COVID-19, evidence of pregnancy or lactation, moribund or not expected to survive more than 24 h, unable or unwilling to deliver lung protective ventilation, or receiving extracorporeal membrane oxygenation. Prior to the patient’s participation, the written informed consent form was signed and dated by the patient or their legally authorized representative, and the person who conducted the informed consent discussion.

### DSMB Review

A DSMB was formed and charter was established. Members of the DSMB were a biostatistician with a focus on cellular therapy, a stem cell expert, and a pulmonologist. The DSMB was notified immediately for all serious or unexpected AEs directly related to the study product.

### hCT-MSC Manufacturing

The Marcus Center for Cellular Cures at Duke University manufactured the study product (allogeneic, hCT-MSC) in the Robertson GMP Cell Manufacturing Laboratory. These hCT-MSC also currently are or have been utilized in clinical trials to treat pediatric patients with autism spectrum disorder, cerebral palsy, hypoxic ischemic encephalopathy, and adults with osteoarthritis of the knee.^9,10^ Manufacturing is identical for all products used in these clinical trials. The final product is a cryopreserved passage 2 (P2) product that is thawed and diluted on the day of infusion.

The hCT-MSCs are manufactured from cord tissue donated to the Carolinas Cord Blood Bank at Duke, an FDA licensed public cord blood bank (DUCORD), by mothers delivering healthy term male babies by Cesarean section after full written informed consent from the newborn infant’s mother. Full donor screening and testing are performed in accordance with regulatory requirements (21 CFR 1271.75, 1271.80, and 1271.85). The cord tissue is harvested in the operating room and placed in a sterile container containing 200 mL of sterile plasmalyte-A solution without antibiotics. The tissue is transported in a validated container at room temperature by a dedicated and trained courier to the manufacturing lab on the same day of the baby’s delivery. In the GMP lab, in the clean room, the cord is cut into 2″ sections and digested with 4 GMP grade enzymes (DNAase, collagenase, alpha hyaluronidase, and papain) on the Miltenyi Octo tissue dissociator (Miltenyi, Bergisch Gladbach, Germany). The resultant cell suspension is plated in a cell stack with XSFM media (Irving Scientific, Santa Ana, CA, USA) supplemented with human platelet lysate (Compass Biomedical, Hopkinton, MA, USA) in a 1-layer cell stack flask (Corning, Glendale, Arizona) and cultured for 7-10 days. When confluent, the cells are harvested (P0) and replated in HYPERFlasks (Corning, Glendale, Arizona) for 7-8 days to confluence and harvested (P1) and then replated again and cultured to P2. The P2 harvest is washed and cryopreserved in a final concentration of 10% DMSO (in PLA/5%HSA) in 5 finger cryobags (Pall Medical), frozen in a controlled rate freezer, and stored in the vapor phase of liquid nitrogen until use. A portion of the P0 and P1 cells are also cryopreserved and stored as part of a master (P0) and working (P1) cell bank during manufacturing.

One P2 dose is thawed and tested for cell count, viability, phenotype, tri-linage differentiation, p53 mutation, maternal cell contamination, sterility (BacT-alert), adventitial virus testing, endotoxin, mycoplasma, and functional assay (suppression of third-party T-cell proliferation). All assays must pass specifications for batch release. Each P2 dose is tested for cell count, viability, and sterility (BacT-alert). All assays must pass specifications for lot release. The post-thaw product is stable for 4 h at room temperature.

### hCT-MSC Dosing and Administration

Ten patients were given 3 doses of hCT-MSCs once a day for 3 days. The dose was 1 million cells/kg (maximum 100 million cells) with ≥70% viability (based on Cellometer [Nexcelom Bioscience], Lawrence, MA), infused over 30-60 min. The second and third infusions of the same number of cells were given 24 h (± 6 h) and 48 h (± 6 h) later, respectively. Treatment was started within 36 h of enrollment. On the day of infusion, the product was thawed, diluted 1:1 with Plasmalyte-A with 5% Human Serum Albumin (HSA), and assessed for total nucleated cell count and viability on a Cellometer. The volume needed for the patient’s dose was then calculated, removed, and diluted in additional Plasmalyte-A with 5% HSA to 40 mL and taken to the bedside for infusion over 30-60 min on an infusion pump.

Patients were premedicated with diphenhydramine (12.5-25 mg IV) and hydrocortisone (0.5 mg/kg IV) prior to hCT-MSC infusion. Patients were monitored with pulse oximetry during and for 1 h after the infusion and then daily thereafter. Vital signs, including BP and respiratory rate, were taken every 15 min during the infusion and every 30 min for 1 h after the infusion. The patients were monitored in the hospital for a minimum of 4 days following the first infusion depending on the resolution of symptoms.

In the event of an infusion-related reaction, eg, generalized urticaria, cough, dyspnea, wheezing, or hypoxemia (pulse oximetry <90%), the infusion was stopped immediately. If medically indicated, the patient was treated with a second dose of diphenhydramine (up to 50 mg IV) and a second dose of steroids (eg, methylprednisolone) (up to 60 mg IV). If these signs or symptoms developed, the infusion was not restarted and the therapy was aborted. If this occurred after the first or second infusion, the subsequent infusion(s) were not administered. If the patient experienced a clinically significant grade 3 or 4 adverse event (AE), defined by the NCI Common Terminology Criteria for Adverse Events (CTCAE) version 5.0, that was considered by the treating physician to be related to the investigational product or due to the infusion procedure, the infusion was stopped and permanently discontinued.^11^ In the setting of grades 1 and 2 reactions, the infusion could be restarted at the treating physician’s discretion if the AE responded to medical management; otherwise, the infusion was discontinued permanently.

### Outcomes

The primary outcome was safety. Safety was evaluated through infusion reactions and adverse event monitoring, including the formation of new HLA antibodies. The secondary outcome was exploring outcomes. Outcomes assessed by (1) survival at 28 days after the first dose of MSCs, (2) increase in PaO_2_/FiO_2_ ratio by 50% by day 3 (72 h after first infusion),^12^(3) days to hospital discharge to home, (4) number of ventilator free days at day 90, (5) 50% decrease in opacities by CT chest 1 week post initiation of MSC therapy, (6) number of oxygen-free days at day 28, and (7) changes in viral load during the first week of treatment with MSCs measured by routine PCR testing. Data collected included clinical assessments, routine laboratory tests, anti-HLA antibodies, lung injury severity, oxygenation, microbiology, and chest imaging at baseline, infusion days (days 1, 2, and 3), day 4, day 7 ± 1, day 14 ± 2, day 28 ± 2, and day 90 ± 5.

#### Adverse Event Monitoring

AEs were assessed on all 3 infusion days, day 4, day 7 ± 1, and day 90 ± 5. An AE was defined as any untoward medical occurrence associated with the use of an intervention in humans, whether or not it was considered to be intervention-related (21 CFR 312.32 (a)). Progression of the patient’s lung disease, or events that were unequivocally due to disease progression, were not reported as an AE (unless it was considered to be related to the investigational product by the treating physician). A serious AE was defined in regulatory terminology as any untoward medical occurrence that resulted in death, was life-threatening, required prolongation of existing hospitalization, results in persistent or significant disability or incapacity, or was an important medical event.

For all collected AEs, the treating physician evaluated the patient to determine the AE’s causality based on temporal relationship and his/her clinical judgment using the following categories: definite—AE was clearly related to the treatment protocol, probable—AE was likely related to the treatment protocol,  possible—AE may be related to the treatment protocol, unlikely—AE is doubtfully related to the treatment  protocol, and unrelated—AE is clearly not related to the treatment protocol. In addition, all AEs were assessed for expectedness. Expected events were those that had been previously identified as resulting from administration of the investigational agent,^13^ with all other AEs considered to be unexpected.

#### Clinical Evaluations

At baseline (after enrollment) the patient’s medical history and demographics were collected. Physical exam results were collected at baseline, all 3 days of infusion, day 4, and day 7 ± 1. Vital signs (blood pressure, heart rate, respiratory rate, temperature, and O_2_ saturation) were recorded at baseline and all 3 days of infusion. Clinical data were collected on other interventions that were expected to affect oxygenation, including use and duration of prone ventilation, neuromuscular blocker administration, inhaled nitric oxide, inhaled epoprostenol, and corticosteroids. Lastly, survival was assessed on day 28 ± 2 and day 90 ± 5.

#### Laboratory Tests

Laboratory test results were collected to monitor safety. Any clinically significant abnormal results observed were entered into the database as an adverse event. Hematology testing was performed at baseline, day 4, and day 7 ± 1. Chemistry laboratory evaluation was performed on baseline, day 4, day 7 ± 1, and day 28 ± 2. Urinalysis was performed at baseline. Coagulation testing was performed at baseline, day 4, and day 28 ± 2. Transfusion transmitted disease screening and HLA typing were performed at baseline. Anti-HLA antibody testing was performed at baseline and day 28 ± 2. COVID-19 PCR was performed at baseline, day 4, day 7 ± 1, and day 28 ± 2.

#### Lung Injury

Lung injury severity was determined by the Murray lung injury score on baseline, infusion days (days 1, 2, and 3), day 4, day 7 ± 1, day 14 ± 2, and day 28 ± 2. The Murray lung score includes findings on chest x-ray, PaO_2_/FiO_2_ ratio, PEEP, compliance (VT/plateau pressure-PEEP). PaO_2_/FiO_2_ ratio was separately recorded on baseline, infusion days (days 1, 2, and 3), day 4, day 7 ± 1, and day 14 ± 2. If not available, the SpO_2_/FiO_2_ ratio was used to impute the PaO_2_/FiO_2_ ratio. Chest x-ray was performed at the discretion of the clinical teams. If a recent radiograph had not been performed, the last one available was used to calculate the Murray score. Arterial blood gas was at the discretion of the clinical team.

### Statistical Analysis

All patients treated were analyzed. Baseline characteristics of the participants were summarized. The number of patients with infusion-related reactions and other unexpected product-related adverse reactions were tabulated descriptively using CTCAE terminology. Continuous outcomes were described using medians and ranges and categorical outcomes were described with counts.

## Results

### Participant Characteristics and Overall Outcome

From August to November 2020, 10 patients (7 females, 3 males) were enrolled in the study at 2 sites (2 Baptist Health, 8 Duke) with a median age of 62.5 years (range 40-79) (Table 1). Patients were dosed with 3 daily doses of hCT-MSC, 1 million cells/kg (maximum total dose 100 million cells) with a viability >70% post-thaw (Table 2). All doses were administered on schedule with no infusion-related reactions and no manufacturing failures. The average cell dose was 0.93 (range 0.56-1.45) cells/kg and the average viability was 85 (range 63-99) %. Five patients were over 100 kg body weight and received 102 (range 85-117) million cells on average. The remaining 5 patients received 86 (range 55-117) million cells. For dosing: 4/30 (13%) doses were less than the study dose of 1 million cells per kg or the maximum dose of 100 million cells, and 29/30 (97%) of the doses met the ≥70% criteria. Five patients died: 3 by day 28 and all 5 by day 90 of the study (median 27 days, range 7-76 days). All deaths were determined to be unrelated to the hCT-MSCs. Transfusion transmitted disease screening (HBV surface antigen, HCV antibody, HIV antibody, HBV core antibody, HTLV types I and II antibody, *Trypanosoma cruzi* antibody, *Treponema pallidum* antibody, cytomegalovirus antibody, HIV-1/HCV/HBV NAT, West Nile Virus NAT, and Zika NAT) were performed at baseline in 9 patients and all were negative, except 1 patient was CMV antibody positive.

**Table 1.** Baseline demographics.

	Participants (*N* = 10)
Age, years	
Mean (SD)	63.2 (12.3)
Median	62.5
Range	(40-79)
Gender (genetic)	
Female	7
Male	3
Race	
Black or African American	2
White	6
Other race	2
Ethnicity	
Hispanic or Latino	3
Not Hispanic or Latino	7
Treating hospital	
Baptist Health	2
Duke	8

**Table 2.** MSC cell dose and viability.

Participant weight, kg (*n* = 9) Median (range)	104 (69-152)
Infusion 1 MSC dose, ×10^6^ cells (*n* = 10) Median (range)	95 (65-116)
Infusion 1 viability, % (*n* = 10) Median (range)	85 (72-97)
Infusion 2 MSC dose, ×10^6^ cells (*n* = 10) Median (range)	90 (55-107)
Infusion 2 viability, % (*n* = 10) Median (range)	84 (63-98)
Infusion 3 MSC dose, ×10^6^ cells (*n* = 10) Median (range)	95 (67-117)
Infusion 3 viability, % (*n* = 10) Median (range)	85 (70-99)

#### Clinical Evaluations

Eight patients, all treated at Duke, received corticosteroids (median duration 9 days [range 5-10 days]), 7 received neuromuscular blockers (median duration 6 days [range 3-17 days]), 4 patients received prone ventilation (median duration 4 days [range 1-16 days]), and no patient received inhaled nitric oxide or epoprostenol (Table 3). In addition, 8 patients received remdesivir, 2 received COVID convalescent plasma and 2 antibiotics.

**Table 3.** Clinical evaluations.

Site	Participant ID	Time to death (days)	Duration of corticosteroid use (days)	Duration of neuromuscular blocker use (days)	Duration of prone ventilation (days)
Duke	1	7	5	4	—
Duke	2	—	8	—	—
Duke	3	39	5	17	6
Duke	4	—	9	—	—
Duke	5	76	10	6	1
Duke	6	27	10	3	—
Duke	7	20	9	7	1
Duke	8	—	6	.	—
Baptist Health	9	—	—	3	—
Baptist Health	10	—	—	16	16
	Median (range)	27 (7.0, 76)	9 (5, 10)	6 (3, 17)	4 (1, 16)

#### Adverse Events

There were no serious or non-serious product or infusion-related adverse events. There were 28 non-serious AEs among 3 unique patients (2, 4, and 22 events in each of the 3 patients), which were all unrelated or unlikely to be related to the study product (Table 4). There we 2 serious AEs (delirium and thromboembolic event) in 2 separate patients, both deemed unrelated and expected.

**Table 4.** Non-serious adverse events.

Summary of adverse events			
**Adverse event**	**Severity**	**Number of events (*N* = 28)**	**Number of subjects (*N* = 3)**
Fever	Level 1—mild	6	2
Ventricular tachycardia	Level 1—mild	4	1
Hypokalemia	Level 2—moderate	3	2
	Level 1—mild	1	1
Sinus bradycardia	Level 1—mild	3	1
Anemia	Level 3—severe or medically significant	1	1
Atrial fibrillation	Level 1—mild	1	1
Edema limbs	Level 1—mild	1	1
Encephalopathy	Level 3—severe or medically significant	1	1
Heart failure	Level 1—mild	1	1
Hyperkalemia	Level 2—moderate	1	1
Hypertension	Level 3—severe or medically significant	1	1
Hypocalcemia	Level 1—mild	1	1
Pneumothorax	Level—mild	1	1
Syncope	Level 3—severe or medically significant	1	1
Urinary tract infection	Level 3—severe or medically significant	1	1

#### Safety

To assess HLA antibody formation, days 0 and 28 HLA antibody testing was performed and was available for 4 patients (2 died before day 28, 2 missing day 0 [1 day 28 positive, 1 day 28 negative], and 2 missing day 28). Three patients had no change in their HLA antibody status: 1 patient was positive and 2 were negative pre- and post-infusion. One patient developed HLA antibodies between day 0 and 28 against HLA A2, A68, A69, and DR15. The HLA type of the hCT-MSC unit was A2, A25, B7, B44, C5, C7, DRB1 04, and DRB1 11. Thus, it appears HLA antibodies were formed against the donor; however, blood transfusion history was not obtained.

#### Laboratory Tests

No abnormal hematology, coagulation, chemistry, or urinalysis testing results were captured under adverse event reporting. All 10 patients were COVID-19 positive at baseline, one patient was tested and negative on days 4, 7, and 28. Another patient tested and positive on day 7.

#### Lung Injury

The Murray lung injury score did not appear to change over the study period; however, not all patients were able to be assessed at each planned time point (Table 5).

**Table 5.** Summary of Murray lung injury score over study period.

	Time point							
Participant ID	Baseline	Infusion day 1	Infusion day 2	Infusion day 3	Day 4	Day 7	Day 14	Day 28
1	3	2.75	2.75		1.75	3	Died	Died
2	2.75	3			2.33			
3	3.75	3.25			3.33	3.33	2.67	3
4	3.67	3.67			4	4		
5	2.75	3			3.5	3.33	3	2.67
6	3	3			3.5	3	2.33	3
7	3.67	3.75	3.75	3.75	4	2.67	4	Died
8	3.25	3.25			3	2.67	3.33	4
9	3	3.33						
10	3.33	3.25			2	3		
Summary								
*N*	10.0	10.0	2.00	1.00	9.00	8.00	5.00	4.00
Mean (SD)	3.22 (0.38)	3.23 (0.31)	3.25 (0.71)	3.75	3.05 (0.84)	3.13 (0.43)	3.07 (0.64)	3.17 (0.58)
Median (min–max)	3.13 (2.75, 3.75)	3.25 (2.75, 3.75)	3.25 (2.75, 3.75)	3.75 (3.75, 3.75)	3.33 (1.75, 4.00)	3.00 (2.67, 4.00)	3.00 (2.33, 4.00)	3.00 (2.67, 4.00)

#### Secondary Outcomes

On day 28, 7/10 (70%) patients were alive. No patients had an increase in PaO_2_/FiO_2_ ratio by 50% by day 3. The median number of days to hospital discharge to home was 19 days (range 8-18), among 3 patients that were discharged alive to home. The median number of ventilator-free days within 90 days was 25.5 days (range 0-80 days). The median number of oxygen-free days within 28 days was 0 days (range 0-21 days) for 9 evaluable patients. No patients had a 50% decrease in opacities on CT chest one week post initiation of MSC therapy.

## Discussion

Our phase I study demonstrated that hCT-MSCs were feasible in patients with COVID-ARDS. All 10 patients  received the 3 IV infusions of hCT-MSCs as scheduled without any infusion-related adverse events. Non-serious and serious AEs as well as participant fatalities were deemed unrelated to the study product. The hCT-MSCs manufacturing and dose preparations demonstrated feasibility. One cell dose thawing preparation out of 30 resulted in viability not meeting criteria, which has subsequently been corrected by (1) increasing training at secondary sites and (2) optimizing our cell counting program with the assistance of the manufacturer.

At the beginning of the COVID-19 pandemic, strategies to treat COVID-ARDS were urgently needed as mortality rates were 50% for mechanically ventilated COVID-ARDS patients.^14^ During the more than 2 years of the pandemic, treatment strategies for COVID-19 and COVID-ARDS were developed that substantially reduced mortality; however, new therapies are still needed.^15^ Treatment with remdesivir and dexamethasone reduced mortality in moderate to severe disease by 32% and 12%, respectively.^16^ Treatment with baricitinib, a JAK1/JAK2 inhibitor, decreased mortality in hospitalized COVID-19 adults by 5%.^17^ Additional treatment with tocilizumab, a monoclonal antibody that inhibits IL-6 receptor, in patients requiring mechanical ventilation decreased mortality by about 5%.^3^ As 1 million people in the United States have died from COVID-19, the use of MSCs or other cellular therapies provides an additional potential treatment option.

MSCs have immunomodulatory and tissue repair capabilities as well as an excellent safety profile in almost 1000 clinical trials treating 10 000 patients.^5^ MSCs can be isolated and expanded from a number of tissue sources, including placenta, adipose tissue, dental pulp, bone marrow, and cord tissue. MSCs must all meet the standard ISCT criteria for quality control, including immunophenotyping, testing for immunosuppressive potential, tumorigenicity  assessment, and evaluation of cellular senescence.^18^ However, studies have demonstrated MSC product variability between donors, tissue source, as well as manufacturing.^19^ hCT- MSCs have the benefits of easier collection, faster proliferation, and potentially lower immunogencity.^19,20^

Multiple clinical trials have investigated the use of allogeneic MSCs in COVID-related severe disease.^5^ These trials have demonstrated that MSCs are safe, decrease inflammatory cytokines resulting from COVID-related cytokine storms, and potentially improve recovery and survival. MSCs potentially hone to the site of injury, which in COVID-ARDS is principally the lung’s alveolar region, and release cytokines and other factors that restore the tissue, reduce T cell, NK cell, and macrophage cytokine release, and clear bacterial infection.^5^ Three randomized controlled trials using hCT-MSCs for COVID-ARDS are published as well as other studies that are underway or use other tissue sources for MSCs.^5,21-23^ In phase I/IIa  double-blind randomized controlled trial using hCT-MSCs in 24 patients with COVID-ARDS, either receiving high-flow oxygen therapy or mechanical ventilation, from April to July 2020, patients in the hCT-MSC group received 2 doses of 100 million cells IV on days 0 and 3 and had improved survival and decreased time to recovery at day 30.^21^ In this study the investigators’ analysis days 0 and 6 plasma concentrations of sTNFR2, TNFα, and TNFβ and demonstrated increased sTNFR2, which inhibits TNFα and TNFβ, and decreased TNFα and TNFβ levels, potentially explaining how MSCs decrease the hyperinflammation resulting in COVID-ARDS.^24^ In another double-blind randomized control trial from May to October 2020 comparing 1 million cells/kg IV hCT-MSCs to placebo in 40 intubated COVID-ARDS patients also demonstrated improved survival in the MSC arm.^23^ In the third ­double-blind randomized controlled trial from April to October 2020 in patients with COVID-ARDS receiving high-flow oxygen therapy or mechanical ventilation, patients received 3 infusions of 1 million cells/kg IV hCT-MSC manufactured from Wharton’s jelly (maximum dose of 80 million cells) or placebo over 5 days.^22^ There were no differences in primary or secondary outcomes of PaO_2_/FiO_2_ ratio change, ventilator duration, organ failure, or mortality. All studies, as well as this study, demonstrated the safety of hCT-MSCs. In addition, these studies used a similar hCT-MSC cell dose to this study and provided 1 to 3 doses. However, there were differences in tissue source (cord tissue versus Wharton’s jelly), tissue donor, MSC cell isolation technique (enzymatic digestion versus explant), culture conditions (media and media supplements), cryopreservation dose and method, passage number (passage 2 versus 5-6, which could decrease proliferation capacity and increase replicative senescence), cell thawing, and final dose preparation methods. Also there were differences in product testing (cell viability, T-cell suppression, sterility, tri-linage differentiation, p53 mutation, maternal cell contamination, and immunophenotype) requirements and methods. Additionally, in this study, the hCT-MSCs that were provided are well characterized and have been used safely in other clinical trials.^9,10^ Lastly, there were differences in the patient inclusion criteria, such as requiring intubation versus also including those on high-flow oxygen as well as changing care of these patients over time and depending on patient location. These differences make it difficult to compare results from these studies and have been shown to impact the MSC product.^2^ Future studies using hCT-MSCs should clearly define the manufacturing process, the passage number of the MSCs, and the product characterization as well as the participant characteristics and outcomes to understand the study and their outcome differences as well as their MSC product differences.

In this study, 1 patient out of 3 patients, who were negative at baseline and had a day 28 sample, developed a positive HLA antibody screen after hCT-MSC administration. hCT-MSCs carry HLA antigens and the cells are not matched to the patient’s HLA type. HLA antibody formation has been reported in other studies of MSCs.^25^ Notably HLA antibodies can be formed as a result of blood transfusion despite leukoreduction.^26^ Given the only corresponding HLA antigen to the HLA antibodies was HLA A2, which is carried on about half of the US population, and the other HLA antigens did not match the hCT-MSC, it is not conclusive if the hCT-MSCs were responsible for the alloimmunization, In our previous study using hCT-MSCs in patients with autism spectrum disorder, who received 1-3 doses, 5/9 patients developed HLA antibodies at 6 months follow up.^10^ HLA antibody formation appeared to increase with increasing hCT-MSC exposure: 1/3 participants formed HLA antibodies after one dose, 1/3 participants formed HLA antibodies after 2 doses, and 3/6 participants formed HLA antibodies after 3 doses. Additionally, there was more HLA antibody formation against 1 of the 3 donor-derived products, which interestingly was the same donor-derived lot the patient in this study  received. Thus, the number of doses a recipient receives and the donor may play a role in HLA antibody formation.

One limitation of this phase I feasibility study was the limited sample size and lack of a control group, resulting in a small and purely descriptive study. Another limitation is the rapid evolution of COVID care during this study as well as rapidly changing COVID-related hospitalization rates, which resulted in differences in the standard of care over the accrual period. The last limitation was missing data. For example, Murray lung injury scores are missing, which is likely due to difficulty obtaining the data to calculate the score on a daily basis. Another example is missing anti-HLA antibody testing data either on day 0 or day 28 due to missed intensive care unit blood sampling, specimen handling, or discharge or death.

In conclusion, hCT-MSCs are feasible and can be successfully centrally manufactured and infused at multiple locations. Future studies are needed to evaluate their safety and efficacy in COVID-19-related ARDS. Additional MSC production optimization is required to increase the yield of MSCs per hCT donation for scalability, such as implementing a bioreactor, modifying the manufacturing conditions, and expanding culture to passage 3, without compromising cell integrity or product safety. Our study demonstrates a potential framework for future studies investigating the use of hCT-MSCs in COVID-19 patients with ARDS in the intensive care unit.

## Data Availability

The data that support the findings of this study are available from the corresponding author upon reasonable request.
